# Preclinical models of congestive heart failure, advantages, and limitations for application in clinical practice

**DOI:** 10.3389/fphys.2022.850301

**Published:** 2022-08-04

**Authors:** Marta Saura, Jose Luis Zamorano, Carlos Zaragoza

**Affiliations:** ^1^ Departamento de Biología de Sistemas, Facultad de Medicina (IRYCIS), Universidad de Alcalá, Madrid, Spain; ^2^ Centro de Investigación Biomédica en Red de Enfermedades Cardiovasculares (CIBERCV), Instituto de Salud Carlos III (ISCIII), Madrid, Spain; ^3^ Departamento de Cardiología, Hospital Universitario Ramón y Cajal (IRYCIS), Madrid, Spain; ^4^ Unidad de Investigación Cardiovascular, Departamento de Cardiología, Universidad Francisco de Vitoria, Hospital Ramón y Cajal (IRYCIS), Madrid, Spain

**Keywords:** congestive heart failure, large animal models, rodent models, myocardial ischemia, myocardial infarction, hypertension, atherosclerosis, embolization

## Abstract

Congestive heart failure (CHF) has increased over the years, in part because of recent progress in the management of chronic diseases, thus contributing to the maintenance of an increasingly aging population. CHF represents an unresolved health problem and therefore the establishment of animal models that recapitulates the complexity of CHF will become a critical element to be addressed, representing a serious challenge given the complexity of the pathogenesis of CHF itself, which is further compounded by methodological biases that depend on the animal species in use. Animal models of CHF have been developed in many different species, with different surgical procedures, all with promising results but, for the moment, unable to fully recapitulate the human disease. Large animal models often provide a more promising reality, with all the difficulties that their use entails, and which limit their performance to fewer laboratories, the costly of animal housing, animal handling, specialized facilities, skilled methodological training, and reproducibility as another important limiting factor when considering a valid animal model versus potentially better performing alternatives. In this review we will discuss the different animal models of CHF, their advantages and, above all, the limitations of each procedure with respect to effectiveness of results in terms of clinical application.

## Introduction

Heart failure (HF) is one of the most challenging public health concerns and affects a significant portion of the population. (Benjamin et al., 2019). CHF is the manifestation in which the left ventricle fails to provide sufficient blood to meet the minimum metabolic demand. In this scenario, congestion is the consequence of neurohormonal and sympathetic nervous system activation to compensate a reduced cardiac output (CO), increasing cardiac filling pressures through chronically elevated left-sided filling pressures, and connected to the right ventricle, resulting in systemic congestion. It can be defined as a set of situations that make the heart dysfunctional including prevention of adequate tissue perfusion. In an attempt to increase blood flow, the sympathetic stimulation induces vasoconstriction of the splanchnic capacitance vessels and vasodilation of the hepatic veins, the main mechanism to increase cardiac filling pressures. This process is associated to venous congestion disorders, that include a significant increase in both central venous pressure (CVP) and intra-abdominal pressure (IAP).

Clinical manifestations of congestion comprise the development of dyspnea, orthopnea, the appearance of abnormal pulmonary sounds, edema and jugular ingurgitation as a mechanism to compensate for a reduced CO. In many cases, the increase in left filling pressure is responded by the pulmonary system, which eventually shunts to the right ventricle, finally resulting in a systemic congestive problem.

The term congestive heart failure (CHF) unifies both the failing heart and congestive behavior, a combination difficult to explore in the clinic at the molecular level. Classical assessment of left-sided filling pressures includes measurement of left ventricle end-diastolic pressure (LVEDP) and pulmonary capillary wedge pressure (PCWP). Elevated LVEDP is the main indicator of left ventricular diastolic dysfunction (Mielniczuk et al., 2007), while PCWP measures left atrial pressure, through insertion of a balloon-tipped catheter (Swan-Ganz catheter) to occlude a branch of the pulmonary artery (Aalders and Kok, 2019). In both cases cardiac catherization is required, and hence, assessing these two parameters are limited to humans and big animal models of CHF. To overcome these limitations, non-invasive assessment of filling pressures are useful alternatives. In this regard, echocardiographic quantification of left ventricle filling pressure is based on E/e´ measurement: the ratio between early diastolic velocity on transmitral Doppler (E) and the early diastolic velocity of mitral valve annulus from tissue Doppler (e´). However, this ratio is still under debate as predictor of left ventricle filling pressure (Sharifov and Himanshu Gupta, 2017). On the other hand, a non-invasive echocardiographic assessment of left atrial (LA) strain is used to evaluate heart failure with preserved ejection fraction to predict disease severity and risk of severe outcome (Reddy et al., 2019). CHF is also assessed by lung ultrasound, and in particular ultrasound lung comets, as to visualize extravascular lung water, is a useful method to estimate pulmonary congestion in heart failure patients (Li et al., 2018). Despite all the invasive and non-invasive ways to assess CHF, a set of molecular fingerprints that can shed light on the early diagnosis and progression of disease is still poorly understood, which implies the need to develop reliable animal models that may help to a better understanding and effective approach to this pathology.

Ideally, a valid experimental model should be reproducible, having to fulfill, if not all, a significant number of the features of disease. Furthermore, translation of the experimental results to the clinic must represent a top priority, and hence, although there is a wide variety of small preclinical models of HF, the use of animals with a cardiovascular system closer to humans, such as swine or sheep models, are preferably used for the generation of valid results. In view of the above, the following are models used with varying degrees of reproducibility (Table 1), thanks to which we can now gain a deeper understanding of this complication.

**TABLE 1:** Preclinical models of CHF.

Preclinical model	Procedure	Advantages	Limitations	Species	References
Myocardial Infarction	External oclusion of cornary artery	Economic cost Sample sizes	Different anatomy and hemodinamics External oclussion of the artery	Rodents	Cuadrado et al. (2016)
			High variability		
			High mortality rates CVP minimally affected		
	Open chest internal oclusion of coronary artery	Similar collateral	Economic cost Sample sizes	Pigs	Heusch et al. (2011), Heusch and Rassaf, (2016)
		Similar anatomy and hemodinamics	Open chest requirement		
			Inflammation related side effects in response to surgery Risk of V-FIB		
	Close chest PCI angioplasty balloon inflation	Similar collateral	skilled trained personnel in percutaneous catheterization Economic costs	Pigs	Heusch et al. (2011); Ishikawa et al. (2014); Hernandez et al. (2021)
		Similar anatomy and hemodinamics			
			Sample sizes Risk of V-FIB		
Microembolization	Injection of microshperes into the LAD and/or LCX	Severe ventricular dysfunction Increase of PCWP	Does not recapitulate human condition Risk of embolic infarction at the LCX	Dog	Franciosa et al. (1980), Ikeda et al. (2001)
		LV dilatation			
		Reduction of LV wall thickness			
	Coronary slow flow model by receiving repeated low-dose LAD microsphere injection	LV remodeling at 4 weeks of procedure	Does not recapitulate human condition Economic costs	Pig	Hu et al. (2014)
			Sample sizes		
	Ischemia/reperfusion plus autologous injection of platelet thrombi	Severe reduction of LVEF and CO Diastolic ventricular failure Increased PCWP	Does not recapitulate human condition Economic costs	Pig	Sassi et al. (2019)
			Sample sizes		
			Risk of V-FIB		
Pacing-Induced Tachycardia	Accelerated chronified atrial and ventricular rhythms with HR between 200–400 bpm	Biventricular dilatation Ventricular dysfunction Neurohormonal stimulation Severe reduction of LVEF and CO	Absence of mechanistic information about the underlying mechanisms that lead CHF	Dog, Pig	Riegger and Liebau (1982); Moe et al. (1989); Ohno et al. (1994)
		Cesation restores hemodynamic values			
Atrial Fibrilation	Applying cycles of atrial pacing	Atrial fibrilation	Lack of manifestations of CHF excepting dogs	Dog, Goat, Pig Mouse	Power et al. (1998); Yarbrough and Spinale (2003); Dosdall et al. (2013) Zhang et al. (2019)
		Dogs recapitulate pathogenesis of disease In rodents, GWAS/CRISPR are used to find new targets of disease			
Aortic Banding	Surgical banding of the ascending aorta	Procedure mirrors aortic stenosis Neurohormonal activation	Elevated mortality high variabiliy intra and inter species	Dog, Sheep, Pig	Dellsperger and Marcus (1990); Moorjani et al. (2003); Wang et al. (2006); Moorjani et al. (2007); Ishikawa et al. (2015); Chaanine et al. (2017); Fleenor et al. (2018); Merino et al. (2018); Hayward et al. (2019); Olver et al. (2019); Baranowski et al. (2021)
		LV dysfunction Depressed LVEF and CO			
		Mithocondrial, Endothelial, microvascular dysfunction HF with preserved LVEF			
		Correlation with cerebrovascular dysfunction in pigs			
Pulmonary Banding	Surgical bainding of the central or in combination with the left pulmonary artery	Right ventricule dysfunction Chronic pressuer overload Development of HF Neurohormonal activation	Variability intra and inter species Absence of peripheral edema	Dog, Pig	Pereda et al. (2014); Wehman et al. (2017); Nguyen-Truong et al. (2020); Ukita et al. (2021a); Ukita et al. (2021b)
			Absence of pulmonary vascular remodeling		
Hypertension	Surgical by embolization of renal arteries	HF with preserved LVEF	Few sample sizes, and studies	Pig	van de Wouw et al. (2021), Sharp et al. (2021)
	in combination of streptozotozine and western diet	Microvascular dysfunction Integration of cormobidites			
Toxicity	Pharmacological be treatment with deoxycosterone and western diat	Dilated cardiomyopathy in dog and sheep Left ventricular dilatation	Variability intra species Partially recovery after 1 week in dogs	Dog, Seep, Rabbit, Rodent	Movahed et al. (1994); Toyoda et al. (1998); Chekanov (1999); Aguero et al. (2016); Hong et al. (2016); Weil et al. (2018); Galán-Arriola et al. (2019); Yip et al. (2020); Yeh et al. (2021)
	Doxorubicin				
	Norepinephrine				

### Myocardial ischemia

Myocardial ischemia often leads to adverse ventricular remodeling and CHF as a multifactorial process in which neurohormonal response, activation of cell death signaling pathways, and persistent degradation of myocardial extracellular matrix play a major role.

The development of ischemic-induced CHF models, including recapitulation of the patient’s underlying condition, is a challenge of great complexity. Etiologically, CHF involves a set of significant events over the life time and is further aggravated by patient´s comorbidities and polymedication (Pound and Ritskes-Hoitinga, 2018). Furthermore, ischemia-induced CHF is biased by factors like the age, sex, ethnic conditions, and even geographic location (Virani et al., 2021). Besides, current models of myocardial ischemia in the absence of subsequent reperfusion are not representative of human disease. Therefore, many animal models have failed, since almost no the aforementioned factors are addressed, and in general models use young, healthy animals, of the same sex and from the same pool (Pound and Ritskes-Hoitinga, 2018).

Several preclinical models have been developed by external occlusion of one or more coronary arteries, which is not representative of the pathophysiology of disease, since intracoronary plaque formation is the leading culprit of disease (Lee et al., 2017). In addition, Although rodents offer substantial advantages over other species: low cost, large sample sizes and relative ease of genetic manipulation, nevertheless, the differences in coronary architecture, cardiac anatomy, and hemodynamics imply the choice of other species as better candidates for the study of CHF, in which the large animal models have provided significant advances for the clinical practice.

### Acute myocardial infarction

Porcine models of AMI are widely used for reasons ranging from similar collateral circulation (Hill et al., 2009) and architecture (Dixon and Spinale, 2009) to humans, making possible to predict, and control infarct size and disease severity.

As with rodent models (Cuadrado et al., 2016), the procedure can be induced by exposing the heart and externally occluding the coronary artery(s) of interest with gradually inflating pressure rings, which ultimately precipitates the development of an AMI and collateral circulation. Other models comprise AMI in dogs, although extensive collateral flow justifies porcine models (Maxwell et al., 1987; Sabe et al., 2016).

Preclinical open-chest models have provided a substantial amount of information to characterize HF in depth, yet they do not recapitulate the pathogenesis of disease, since intracoronary atherothrombotic lesions are the most frequent cause leading to tissue necrosis, and the inflammatory response related to open-chest surgery, consequently limit the clinical translation.

Currently, percutaneous catheterization by angioplasty balloon inflation and reperfusion, closely resembles interventional catheterization, and is by far the most commonly preclinical model of AMI (Heusch et al., 2011; Hernandez et al., 2021). The left anterior descending coronary artery (LAD) is occluded in most models given the larger size of MI and induces more severe systolic dysfunction. The left circumflex coronary artery (LCX) can also be occluded to induce mitral regurgitation, but LAD occlusion causes more severe left ventricle (LV) remodeling than LCX occlusion (Ishikawa et al., 2014). Limitations include the need of a surgical room, and skilled trained personnel in percutaneous catheterization. In addition, pharmacological support to avoid arrhythmogenic behavior, and sometimes ventricular fibrillation (V-FIB) cardioversion procedures are mandatory in many cases. However, the procedure also offers to perform cardiac preconditioning, the event that occurs when patients undergo coronary occlusion and reperfusion in short and repeated times, prior to a longer temporary occlusion, a mechanism previously described of myocardial cardioprotection (Heusch and Rassaf, 2016), and reduced arrhythmogenic behavior (Ramirez-Carracedo et al., 2020). In addition, if percutaneous occlusion of the coronary artery last no longer than 60 min part of ischemic tissue remains contractile (myocardial salvage), allowing to perform studies of cardiac protection (Santos-Gallego et al., 2016). However, in case of HF studies, at least 2 hours of coronary occlusion are mandatory (Santos-Gallego et al., 2019). These models of HF also allow for investigation of myocardial metabolism either by PET or with direct trans cardiac gradient of metabolites by simultaneous sampling of coronary artery and coronary sinus (Christensen et al., 2017; Santos-Gallego et al., 2019).

### Microembolization

Angioplasty balloon inflation is a noninvasive procedure that partially recapitulates the occlusion that defines acute anginous behavior. However, in most situations, the patient suffers a progressive coronary artery occlusion over time that eventually leads to AMI, in which cardiac remodeling and thus CHF, has already begun and progress ahead of infarction. A canine microembolization procedure initially emerged by injecting microspheres into the LAD and/or LCX artery, over time (Franciosa et al., 1980), while injection of 5 separate dosages every 2 weeks in sheep resulted in severe ventricular dysfunction (left ventricle ejection fraction, LVEF <25%), together with a marked increase in PCWP and dilatation of the left ventricle, accompanied by a significant reduction in left ventricular wall thickness, indicative of CHF (Ikeda et al., 2001). As an important concern, the thrombus generated by using microspheres does not fully resemble human condition, and microembolization of the LAD, can often result in embolic infarction in the LCX.

Patients with slow-flow after percutaneous coronary intervention have a worse prognosis after revascularization. However, the studies were limited to ischemia/reperfusion, which partially reflects this condition, since no- or slow-flow is induced by microembolization. To this regard, an angiographic coronary slow flow model was created in pigs receiving repeated low-dose LAD microsphere injection up to a total of about 300,000 microspheres, in which left ventricle remodeling was visible even 4 weeks after post-procedure (Hu et al., 2014). More recently, the combination of coronary ischemia/reperfusion together with microembolization from autologous injection of platelet thrombi in pigs, provided a new model of CHF, with significant decreases LVEF and CO after 1 week of embolization, together with the appearance of diastolic ventricular failure and increased PCWP (Sassi et al., 2019).

### Tachyarrhythmias

Dilated cardiomyopathy (DCM) is often associated with CHF in response to sustained arrhythmogenic complications over time, like atrial fibrillation (AF), which increase up to three fold the risk of HF (January et al., 2014). In this regard, models should recapitulate ventricular wall dilatation and thinning, along with a progressive decline in cardiac function, and neurohormonal activation.

### Pacemaker-induced tachycardia pacing-induced tachycardia

Over the years, different models of PIT have been developed in dogs, sheep and pigs that eventually lead to reduced ejection fraction. Accelerated chronified rhythms, both atrial and ventricular, with heart rates ranging between 200 and 400 bpm, can generate eccentric bi-ventricular dilatation, inducing ventricular dysfunction and neurohormonal stimulation, accompanied by a significant reduction in LVEF and CO (Riegger and Liebau, 1982). Interestingly, cessation of pacemaker activation returns hemodynamic values to physiological levels. However, the procedure does not shed light on the underlying mechanisms leading to CHF, as heart failure in patients precedes arrhythmogenic behavior (Ohno et al., 1994).

### Atrial fibrillation

As mentioned above, AF may also lead to CHF. In response to a significant reduction of stroke volume, AF patients develop adverse cardiac remodeling, neurohormonal activation and, at the end, heart failure. AF is also a very complex arrhythmogenic disease in regard to its etiology, which limits the development of reliable AF-induced CHF models. The advantages of using pigs lie in the similarity of cardiac architecture, coronary architecture and electrophysiology with humans. However, the substantial risk of developing episodes of V-FIB should be considered before using this species. Indeed, animal models consisting on applying several cycles of atrial pacing in sheep (Moe et al., 1989), dogs, goats and pigs (Yarbrough and Spinale, 2003; Dosdall et al., 2013) have tried to reproduce AF-dependent CHF, but only dogs partially recapitulated the pathogenesis of disease.

Rodent models have recently become a significant advance in the study of AF, thanks to the burst of genetic data through GWAS (Genome-wide association study). In combination with CRISPR/Cas9, many difficult-to-detect non-coding targets implicated in this pathology have been found in mice, which may shed light about the molecular mechanism of disease (Zhang et al., 2019). However, the association with CHF in rodent models is still poorly understood.

### Pressure overload

CHF is also related to chronic pressure overload. Continually having to overcome elevated afterload values eventually reverts to adverse remodeling, where patients develop left ventricular hypertrophy to maintain adequate perfusion, along with increased myocardial stiffness leading to increased ventricular filling pressures and pulmonary congestion. For this reason, patients transiently undergo to an interesting phenotype similar to heart failure with preserved LVEF (Braunwald, 2018; Silva and Emter, 2020). As seen below, large animal models of PO have been developed with CHF symptoms that include pulmonary congestion or dyspnea.

### Transverse aortic constriction (aortic banding)

Aortic constriction models aim to recreate by surgical techniques the phenomenon of aortic stenosis through vessel constriction. This procedure mirrors aortic stenosis for up to 4 weeks depending on stenosis diameter (Merino et al., 2018), which locally triggers neurohumoral activation along with increased myocardial afterload, resulting in increased left ventricular pressure gradient, left ventricular hypertrophy, and myocardial stiffness.

Initially developed in dogs (Dellsperger and Marcus, 1990), aortic constriction in sheep lead to HF, with depressed LVEF, whereas in swine models left ventricular stiffness is the first manifestation (Ishikawa et al., 2015), in which PO is generated by aortic constriction for months (Moorjani et al., 2003; Wang et al., 2006; Moorjani et al., 2007; Fleenor et al., 2018). In these models, the neurohumoral response plays a key role, conditioning adverse remodeling through activation extracellular matrix proteolytic enzymes, together with mitochondrial, endothelial, and microvascular dysfunction (Hayward et al., 2019). However, although constriction devices apply gradual increases in afterload, it takes years for patients to reach the same level of complication, and mortality rates are lower compared to animals. Notably, in almost all species tested, a common feature is the HF with preserved LVEF, which, considering the enormous importance of this pathology yet to be fully explored, makes these models of current relevance and interest (Chaanine et al., 2017; Olver et al., 2019).

Aortic constriction in combination with a high fat diet, have pointed the evidence between the association of HF with cerebrovascular failure, neuroinflammation and amyloidosis with preserved LVEF. At the molecular level and considering limitations that include a lack of nutritional control groups and histopathological data, a transcriptomic pattern can be obtained in animals subjected to aortic constriction typical of frontotemporal dementia and Alzheimer’s disease, implying the clear relationship between HF with preserved LVEF with cognitive impairment (Baranowski et al., 2021).

### Hypertension in combination with other factors

It is increasingly common to identify HF with preserved LVEF, although its etiology, progression and therapeutic targets are largely unknown. In this regard, systemic PO models have been developed in combination with streptozotocin-treated diabetic pigs undergoing renal artery embolization and fed a hypercholesterolemic diet to create a clear model of HF with preserved LVEF and microvascular dysfunction (van de Wouw et al., 2021). More recently, a new model of HF with preserved LVEF has been developed, integrating most of the comorbidities of this pathology, exhibiting sensitivity to obesity, metabolic syndrome, and vascular disease, through a combination of hypercholesterolemic diet and treatment with deoxycorticosterone as a hypertensive agent. As commented by the authors, the main limitation is that conclusions are based on a very small sample size (Sharp et al., 2021), and therefore, more testing is required for further validation of the model.

### Pulmonary artery constriction (pulmonary banding)

Constriction of the pulmonary artery have been used in different animal species to develop a chronic pressure overload model that ends in right HF, a good approach for the study of CHF, since increases right ventricular and central venous pressures.

Although rodent studies are commonly used, the clinical interest in CHF made possible to generate different experimental models in large animals, including two recent studies in which the central pulmonary artery is constricted in sheep (Nguyen-Truong et al., 2020) or simultaneously combined with ligation of the left pulmonary artery (Ukita et al., 2021a). More recently, the use of porcine models of pulmonary hypertension by pulmonary vein constriction (Pereda et al., 2014; Ukita et al., 2021b), has evidenced its validity for studying HF in pathologies related to regenerative cardiology (Wehman et al., 2017). These models can also be used to evaluate the efficacy of therapeutic interventions such as the effect of intratracheal delivery of SERCA2a to ameliorate chronic post-capillary pulmonary hypertension by using a porcine model of chronic post-capillary PH by partial pulmonary vein banding (Aguero et al., 2016).

As we have described throughout this review, a series of surgical approaches have helped us to better understand the etiology and progression of CHF, providing new molecular targets and novel therapeutic approaches to increase the life quality of patients. In addition, it should also pay attention to pharmacology, as a useful strategy in specific aspects not to improve, but to induce HF models, as we will discuss below.

### Toxicity models

Among the different substances affecting cardiac performance, the role of doxorubicin as a chemotherapeutic agent is of particular interest, since it is noteworthy that it often leads to the development of dilated cardiomyopathy in a significant number of cases. The search for new molecular targets against the impact of doxorubicin is subject of intense research, having initially developed preclinical models in dogs (Movahed et al., 1994; Toyoda et al., 1998), sheep (Chekanov, 1999) and more recently in pigs (Galán-Arriola et al., 2019). However, the bulk of research is currently being conducted in rodent (Yip et al., 2020; Yeh et al., 2021) and rabbit models (Hong et al., 2016), with the aforementioned limitations derived from the use of small animal models. The relationship between neurohormonal overproduction of catecholamines and heart failure led to development of models of high norepinephrine exposure in pigs (Weil et al., 2018). Although increasingly out of use, dogs exposed to catecholamines have been developed signs of CHF. Prolonged norepinephrine infusion should be investigated for translation to the clinic, as the model may not be directly comparable, as claimed by the authors, because the norepinephrine infusion was relatively brief (Movahed et al., 1994).

## Conclusions

Congestive heart failure is a highly complex pathology from its onset at the subclinical level, in symptomatology and prognosis. Therefore, it is key to tackle CHF from different perspectives in order to find new molecular targets that enable a significant improvement against this pathology. In this regard, preclinical models of CHF, particularly large animal models, play a central role in providing new tools for early diagnosis and treatment. Although economic costs, handling, personnel skills, and the necessary equipment are often limiting factors, large animal models offer important advantages in terms of better clinical translation: they offer greater structural and functional similarity, and in general many models recapitulate the associated comorbidities. Their use also adds valuable information about safety of existing treatments, even adding economic value in terms of patient outcome.
