# Corrigendum: Preclinical models of congestive heart failure, advantages and limitations for application in clinical practice

**DOI:** 10.3389/fphys.2022.1045550

**Published:** 2022-10-10

**Authors:** Marta Saura, Jose Luis Zamorano, Carlos Zaragoza

**Affiliations:** ^1^ Departamento de Biología de Sistemas, Facultad de Medicina (IRYCIS), Universidad de Alcalá, Madrid, Spain; ^2^ Centro de Investigación Biomédica en Red de Enfermedades Cardiovasculares (CIBERCV), Instituto de Salud Carlos III (ISCIII), Madrid, Spain; ^3^ Departamento de Cardiología, Hospital Universitario Ramón y Cajal (IRYCIS), Madrid, Spain; ^4^ Unidad de Investigación Cardiovascular, Departamento de Cardiología, Universidad Francisco de Vitoria, Hospital Ramón y Cajal (IRYCIS), Madrid, Spain

**Keywords:** congestive heart failure, large animal models, rodent models, myocardial ischemia, myocardial infarction, hypertension, atherosclerosis, embolization

In the published article, the Funding statement was omitted by mistake. The correct Funding statement appears below.

“Proyectos de i+D+I, from the program Investigación orientada a los retos de la sociedad, cofounded by Fondo Europeo de Desarrollo Regional (FEDER) A way to achieve Europe (MINECO/AEI/FEDER/EU SAF2017-87342-R)”, and “PDC2021-121817-I00 cofounded by MICIN/AEI/10.13039/501100011033 and European Union Next GenerationEU/PRTR”.

The authors apologize for this error and state that this does not change the scientific conclusions of the article in any way. The original article has been updated.

